# Gastrointestinal Hemorrhage Secondary to Duodenal Cystic Dystrophy in Heterotopic Pancreas

**DOI:** 10.4021/gr257w

**Published:** 2011-01-20

**Authors:** Alicia Brotons, Maria Dolores Pico, Javier Sola-Vera, Carlos Sillero, Amador Cuesta, Israel Oliver

**Affiliations:** a Department of Gastroenterology, Hospital General Universitario de Elche, Alicante, Spain; b Department of Surgery, Hospital General Universitario de Elche, Alicante, Spain

**Keywords:** Cystic dystrophy, Heterotopic pancreas, Hemorrhage, Paraduodenal pancreatitis

## Abstract

Cystic dystrophy of the duodenal wall (CDDW) is a complication of heterotopic pancreatic tissue located in the wall of the gastrointestinal tract, characterized by the presence of multiple small cysts, usually found in the wall of the second part of the duodenum. Gastrointestinal hemorrhage due to CDDW is a rare complication. We report the case of a 50-year-old man who was admitted to our hospital for persistent vomiting. The imaging tests confirmed the diagnosis of CDDW. During his stay in hospital, the patient had a gastrointestinal hemorrhage secondary to this disorder, which made it necessary to perform a Roux-en-Y gastrojejunostomy (Billroth III).

## Introduction

The term paraduodenal pancreatitis is used to define a different kind of chronic pancreatitis characterized by recurrent inflammatory changes, similar to those seen in chronic pancreatitis, which affect ectopic pancreatic tissue found in the wall of the digestive tube, usually in the pancreatoduodenal groove. There are different types of paraduodenal pancreatitis. The differences reside in the form of presentation, with groove pancreatitis and cystic dystrophy of the duodenal wall (CDDW) being the most frequent. Other less frequent types are duodenal myomatosis, characterised by the presence of smooth muscle around the ectopic pancreatic ducts, and pancreatic hamartoma of the duodenum, characterised by a typical distribution of the acini and ducts, together with stromal proliferation.

Groove pancreatitis is characterised by the presence of fibrosis in the area of the pancreatoduodenal groove. This type of pancreatitis causes great difficulty in the differential diagnosis with adenocarcinoma of the pancreas since on imaging tests the presence of a mass adjacent to the head of the pancreas is seen. Its most frequent clinical features are abdominal pain and duodenal and/or biliary obstruction.

CDDW is defined by the presence of multiple small cysts located in the wall of the digestive tube. We describe the case of a patient admitted to our hospital for persistent vomiting. Based on imaging tests, CDDW was diagnosed. During his stay in hospital the patient suffered an episode of gastrointestinal hemorrhage secondary to intracystic bleeding, which eventually made it necessary to perform a Roux-en-Y gastrojejunostomy.

## Case Report

A 50-year-old man reported vomiting after meals, anorexia, loss of 14 kg of weight, abdominal pain and fullness for the previous two months. Of interest in his personal history was the fact that he had essential thrombocytosis, with secondary intrahepatic portal thrombosis, which was being treated by the hematology service with hydroxyurea (500 mg a day) and sodium dalteparin (7500 UI daily). The patient reported consuming approximately 60 g of alcohol a day and smoking 20 cigarettes a day.

On physical examination, there was slight pain in the epigastrium on palpation of the abdomen. Blood analysis gave the following results: glucose 111 mg/dl (75 - 110), total protein 6.28 g/dl (6.60 - 8.30), albumin 2.07 g/dl (3.50 - 5.20), prealbumin 9.3 mg/dl (20 - 40)**,** amylase 96 U/L (22 - 80), lipase 119 U/L (21 - 67), total bilirubin 0.68 mg/dl (0.30 - 1.20), GOT 16 U/L (10 - 39), GPT 15 U/L (10 - 45), GGT 37 U/L (10 - 55), FA 85 U/L (30 - 120), hemoglobin 15.6 g/dl (13 - 17), leucocytes 12,200/µl (4,000 - 11,000), platelets 830,000/µL (130,000 - 450,000). The tumor marker levels were normal: alpha fetoprotein 1.30 UI/ml (0 - 5), carcinoembryonic antigen 2.83 UI/ml (0 - 5) and CA 19-9 8.27 UI/ml (0 - 37).

**Figure 1:**
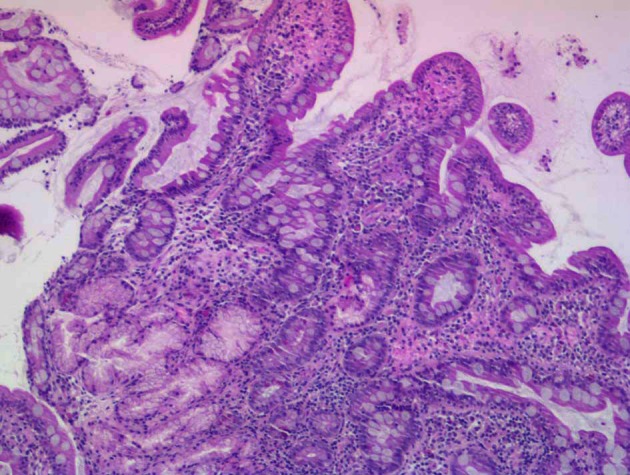
Mucosa with normal architecture. Chronic lymphoplasmocytic inflammation is seen in the lamina propia (staining with hematoxylin - eosin).

Gastroscopy showed a stomach which retained food; at the level of the duodenal bulb there were edematous folds and stenosis, through which the endoscope could pass. Directed biopsies revealed a mucosa of normal architecture, with no villous atrophy. Chronic lymphoplastocytic inflammation was seen in the lamina propia with no acute activity or atypical changes (Fig. 1).

**Figure 2:**
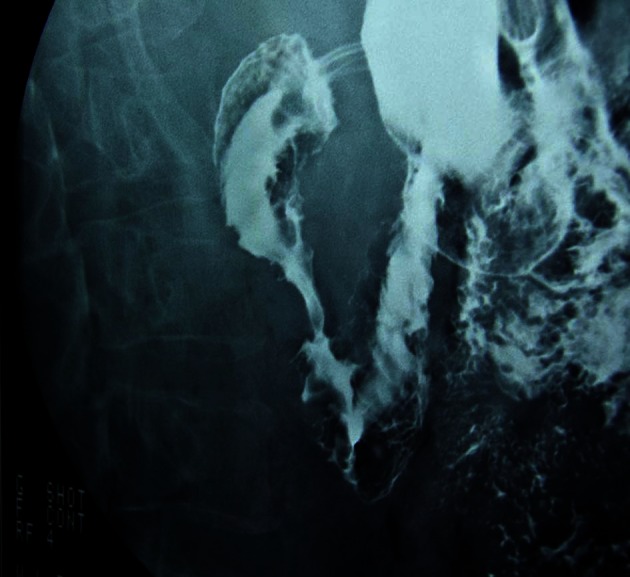
Gastric and duodenal dilatation is seen prior to stenosis in the second part of the duodenum.

**Figure 3:**
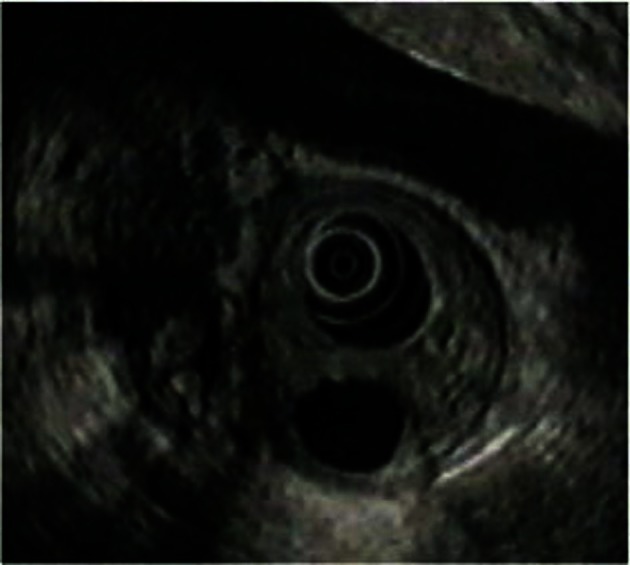
Cyst and thickening of duodenal wall on echoendoscopy.

In view of these findings, an intestinal barium follow-through was requested, which confirmed the delay in gastric emptying (Fig. 2). Computerized tomography and echography showed parietal thickening of the first part of the duodenum, with stenosis of the lumen at this level and gastric dilatation. No structural alterations were seen in the pancreas. On abdominal magnetic resonance a tumor measuring 4 cm was seen near the pancreatic parenchyma, at the level of the duodenal curve, which stenosed its lumen. Finally, echoendoscopy was requested which showed a permeable stenosis of the duodenal bulb and a slight thickening of the duodenal wall. Likewise, several cysts were seen within it, the largest of which measured 1.4 x 0.5 cm. The head of the pancreas had a heterogeneous pattern but no focal lesions were seen, although there were two images of 1 cm twisted in appearance due to dilatation of the pancreatic ducts (Fig. 3).

During his stay in hospital the patient’s condition worsened due to a progressive deterioration in his nutritional status, which required parenteral nutrition, and due to an episode of gastrointestinal hemorrhage, associated with hemodymanic instability, which required supportive measures (intravenous fluid therapy, blood transfusion) and emergency gastroscopy. The gastroscopy showed at the level of the first part of the duodenum and the proximal portion to the second part, in the area of the stenosis described above, an erythematous mucosa, friable, ulcerated and with adherent blood clots. No active bleeding or visibly bleeding vessel was found, and so no endoscopic therapeutic measures were taken. No lesions were found distal to the stenosis.

Following the endoscopy, the clinical condition of the patient continued to deteriorate due to persistence of gastrointestinal bleeding, with hemodynamic instability, which required further blood transfusions. Finally, the surgery service was consulted and it was decided to perform a Roux-en-Y gastrojejunostomy (Billroth III) in view of the patient’s critical state due to his state of malnutrition and precipitated by the episode of gastrointestinal bleeding. The post-surgical course was favorable, the symptoms of duodenal obstruction disappeared completely, the patient was eating adequately and the gain in weight was appreciable.

## Discussion

CDDW is defined as the presence of multiple cysts, usually smaller than 1 cm, associated with inflammatory changes and fibrosis, which cause thickening of the gastrointestinal wall. Their origin resides in the presence of ectopic pancreatic tissue in the wall of the digestive tube, and they present no anatomical or vascular continuity with the pancreas. These inflammatory changes are usually the result of episodes of recurring pancreatitis that affect the ectopic pancreatic tissue. They are often found in the second part of the duodenum (especially around the minor papillar), and less frequently in the stomach and jejunum. In anatomopathological studies, the submucosa and intrinsic muscle that surround the cysts show fibrous and inflammatory changes, similar to those seen in chronic pancreatitis. Dilatation of multiple ducts in the duodenal wall are seen as well as a thick secretion inside it, with pseudocystic changes and adjacent stromal reaction, hyperplasia of Brunner’s glands, dense stromal myoid proliferation and excessive fibrosis [1].

The physiopathology of this condition is still not clear. There are two theories that may explain its occurrence. According to one theory it is secondary to obstruction of the small ducts of the exocrine lobules of the heterotopic pancreatic tissue, giving rise to recurrent episodes of pancreatitis [2]. The other proposed mechanism involves the toxic effect of alcohol on heterotopic pancreatic tissue, as occurs in the pancreas [3], since in most series almost all these patients are seen to have a history of high alcohol consumption.

The incidence of this entity varies from 1% - 14% of the necropsies performed [4] and mainly affects men in their fifties. Its course is usually asymptomatic. When there are symptoms, the most common ones are vomiting and jaundice secondary to obstruction of the duodenum and bile duct, respectively.

Gastroscopy and gastroduodenal transit may confirm the existence of duodenal obstruction. Computerized tomography and magnetic resonance are useful to evaluate the presence of cysts inside the thickened duodenal wall. In some cases changes in the pancreatic paranchyma and/or main pancreatic duct or calcifications may be seen.

In various radiological studies, cysts in the wall of the gastrointestinal tube were detected using CAT in 70% of the patients [5] and in another series in 95% of the patients [6]. They are multiple and small in size. Although the majority are found in the second part of the duodenum, they are also sometimes found in the pylorus or in the first part of the duodenum. However, the most useful imaging test for diagnosing this condition is echoendoscopy, since the sensitivity and specificity are increased to as much as 86%. With this technique it is possible to see the parietal thickening of the inside edge of the second part of the duodenum secondary to the presence of cystic formations. Furthermore, it is useful to evaluate the extent and site of the inflammatory changes [7]. A hypoechoic image between the duodenum and pancreas is characteristic, together with thickening of the duodenal wall and changes in the calibre of the bile duct and canal of Wirsung.

Gastrointestinal hemorrhage secondary to CDDW is a very rare complication and although it is a condition whose diagnosis is improving, thanks to the wider use of echoendoscopy, in our review of the bibliography we found only two articles that had described this complication. One of them described a retrospective series in which this complication was reported in 5% of the cases [8]. In the other study, the case of a 15-year-old boy who presented a gastric hemorrhage secondary to intracystic bleeding due to arteriolar erosion caused by cystic fluid and subsequent fistulization of the gastric wall was reported [9]. In our case, the patient also had a gastrointestinal hemorrhage during his stay in hospital secondary to ulceration of the mucosa caused by intracystic bleeding, which eventually required surgery given the persistence of bleeding.

There is controversy as to the treatment of choice in CDDW, but what appears to be clear is that each case should be treated on a case-by-case basis. Medical treatment consists of measures to alleviate the symptoms by reducing the pain, such as fasting, alcohol abstinence and aspiration of gastric contents, use of octeotride or endoscopic drainage. These last two treatments have various disadvantages, the most serious of which is the short duration of their effects [10, 11]. In the case of octeotride its effect is delayed, lasts for a very short time and is not without adverse effects. Endoscopic drainage is reserved for patients with a small number of large, superficial cysts in the duodenal wall. Therefore, this treatment may only be used in a reduced number of patients since in most cases there are numerous, small cysts associated with thickening of the duodenal wall. Moreover, the rate of recurrence following endoscopic drainage is high. The treatment of choice in symptomatic cases, with duodenal and/or biliary stenosis, pain or gastrointestinal bleeding, that do not improve with medical treatment is duodenopancreatectomy. Despite being an aggressive technique, it is more effective in the long term as the area involved is resected. Finally, intestinal bypass procedures are reserved for cases in which there is greater surgical risk. In our case, we opted for this type of surgery due to the poor nutritional status of the patient, aggravated by the episode of gastrointestional hemorrage which could not be controlled with medical treatment.

In conclusion, gastrointestinal hemorrhage secondary to cysts in the intestinal wall of patients with CDDW is a rare complication but one that is difficult to manage with medical treatment. Therefore, in most cases a duodenopancreatectomy is finally done since this entails resection of the segment involved and hence controls the gastrointestional hemorrage and the symptoms of obstruction and pain. In cases in which the clinical situations are poor, however, surgical bypass procedures are opted for.
